# Evolution of a Search: The Use of Dynamic Twitter Searches During Superstorm Sandy

**DOI:** 10.1371/currents.dis.de9415573fbf90ee2c585cd0b2314547

**Published:** 2014-09-26

**Authors:** Sara Harris Smith, Kelly J. Bennett, Alicia A. Livinski

**Affiliations:** GAP Solutions Inc., Contractor Supporting Office of Emergency Management, Division of Fusion, U.S. Department of Health and Human Services, Washington, DC, USA; Office of Emergency Management, Division of Fusion, U.S. Department of Health and Human Services, Washington, DC, USA; National Institutes of Health, Office of Management, Office of Research Services, Division of Library Services, Bethesda, MD, USA

## Abstract

Background: Twitter has emerged as a critical source of free and openly available information during emergency response operations, providing an unmatched level of on-the-ground situational awareness in real-time. Responders and survivors turn to Twitter to share information and resources within communities, conduct rumor control, and provide a “boots on the ground” understanding of the disaster. However, the ability to tune out background “noise” is essential to effectively utilizing Twitter to identify important and useful information during an emergency response. Methods: This article highlights a two-prong strategy in which the use of a Twitter list paired with subject specific Boolean searches provided increased situational awareness and early event detection during the United States Department of Health and Human Services (HHS), Office of the Assistant Secretary for Preparedness and Response (ASPR) response to Superstorm Sandy in 2012. To maximize the amount of relevant information that was retrieved, the Twitter list and Boolean searches were dynamic and responsive to real-time developments, evolving health threats, and the informational needs of decision-makers. Conclusion: The use of a Twitter list combined with Boolean searches led to enhanced situational awareness throughout the HHS response. The incorporation of a dynamic search strategy over the course of the HHS Sandy response, allowed for the ability to account for over-tweeted information, changes in event related conversation, and decreases in the return of relevant information.

## Introduction

**The Use of Twitter in Emergency Response Operations**

Twitter has emerged as a critical source of information in emergency response operations. Following and reviewing publicly available information posted on Twitter in real-time provides an unmatched source of on-the-ground situational awareness. Situational awareness is defined as “all knowledge that is accessible and can be integrated into a coherent picture, when required, to assess and cope with a situation.”^1^ The open flow of information from local government officials, news outlets, and local residents has helped to change emergency response operations and communications. When combined with traditional data sources, Twitter and other forms of open-source information (e.g., YouTube, Flickr, blogs) can complement official reporting to help provide timelier and more complete and robust situational awareness.^2^

The micro-blogging site Twitter, created in 2006, allows users to share information, ideas, and opinions in 140 characters or less. Over the past 8 years, Twitter has grown to more than 241 million active monthly users who send 500 million tweets each day.^*^ 3 As Twitter is open to the public and easily searchable, it is an ideal social media tool for helping to provide situational awareness. Further, several companies have developed freely accessible web-based tools that bring in Twitter data and allow it to be searched and analyzed.

The effectiveness of Twitter as a tool to provide situational awareness to emergency responders was demonstrated in multiple large scale disasters including typhoons,^4^ floods,^5^ wildfires,^6^ and the 2013 Boston Marathon bombings.^7^ Following the 2009 Red River flood in the United States (US), Palen and colleagues^5^ analyzed the use of Twitter, and found that local residents who generally tweeted on a regular basis before the flood, began to tweet almost exclusively about flood-related matters during the flood. For those weeks, the flood *was *their everyday life and became what they tweeted about most, whether about sandbagging efforts, shelter locations, or the needs of displaced families. As the situation subsided and water levels fell, residents returned to tweeting about other aspects of their lives and did not focus exclusively on the flood.^5^ This is the type of local reporting of the current situation that could provide essential situational awareness to emergency workers responding to the flooding. Listening to residents living within the impacted community can result in an understanding of the most urgent needs of the community, and an indication of when the community may be moving towards recovery after the immediate impact of a disaster.

To complement official data sources and reporting, the US Department of Health and Human Services (HHS) Office of the Assistant Secretary for Preparedness and Response (ASPR) started monitoring Twitter for situational awareness in 2009 during the H1N1 influenza pandemic. Since then, ASPR has continued to refine its monitoring techniques and has increased the number of analytic tools used to bring further depth and detail to maintaining situational awareness.

Presently, Twitter is used for supplementing situational awareness in two distinct ways. The first way is by following long-term trends to identify spikes in Twitter conversation that might indicate an emerging public health concern. This form of monitoring is primarily used for long term epidemiological surveillance of diseases such as Middle East Respiratory Syndrome Coronavirus (MERS-CoV) or H7N9 influenza, and requires the use of a Twitter analytics tool that can access historical Twitter data. The second way ASPR monitors Twitter, which is the focus of this article, is event-specific real-time monitoring. In contrast to the long term trend analysis which focuses on identifying deviations from the historical baseline, event-specific real-time monitoring focuses on finding reports on Twitter that provide situational awareness during an emergency response. The search techniques employed by ASPR for monitoring Twitter are meant to filter down to the most relevant information shared in the midst of a disaster. This article will outline the strategy ASPR employed for event-specific real-time Twitter monitoring during the HHS response to Superstorm Sandy in 2012.

## Background

**The Sandy Basics**

Sandy made landfall in the US on the coast of Brigantine, New Jersey as a post-tropical cyclone on October 29, 2012.^8^ ^9^ By meteorological standards, the storm was no longer a hurricane at the time of landfall, but the enormous size of the weather system resulted in the unofficial coining of the term “Superstorm.” This immense storm resulted in catastrophic storm surge along the New Jersey and New York coastlines. Sandy affected 24 states from Florida to Maine with the greatest impact falling on populations within New York, New Jersey, and Connecticut. An estimated $50 billion in damage was inflicted in the US thereby making Sandy the second-costliest storm to hit the US. The storm caused 147 direct deaths, 72 of which occurred in the Mid-Atlantic and Northeast regions of the US.^9^

Under the National Disaster Response Framework,^10^ HHS is tasked with supplying emergency support functions for responses related to public health, medical surge (including patient movement and behavioral health services), and potential mass fatality management. Superstorm Sandy required a substantial emergency response for HHS. Throughout the response, more than 2,300 representatives and officers from the HHS National Disaster Medical System (NDMS) and U.S. Public Health Service (USPHS) Commissioned Corps were deployed, aiding with more than 6,700 patient encounters at hospitals and shelters in New York and New Jersey.^11^

As the storm ended and survivors began to assess the damage from the storm, Twitter was inundated with information. Between October 27, 2012 and November 1, 2012 Twitter users sent more than 20 million tweets using the terms*Sandy, hurricane, #Sandy, *and* #hurricane.* 12 On October 29^th^, in the immediate hours post-landfall, 20% of all searches conducted on Twitter were Sandy-related searches.^13^ Responders and survivors turned to Twitter to publicly share information within communities, conduct rumor control, and share resources. Sandy represented an unprecedented use of social media by responders and survivors alike.

ASPR began monitoring Twitter for indications and warnings of potential public health emergencies as Sandy made landfall in New Jersey on October 29, 2012. For the initial two weeks of the HHS response, Twitter was monitored 16 hours a day every day. The monitoring team consisted of one full-time social media lead analyst (SHS) and ad-hoc ASPR and HHS employees and interns. When possible, monitoring was split into eight hour shifts to make the work load more manageable. Throughout a shift a single analyst would be entirely dedicated to monitoring Twitter searches and lists

During the first two weeks of the HHS response, in addition to monitoring for early reports of public health emergencies, an overview of the trends observed on Twitter was provided for overall situational awareness during twice daily HHS briefings. On November 17, 2012, monitoring was scaled back to regular working hours and daily situational awareness reports were reduced to once a day. ASPR stopped daily monitoring of Twitter for public health situational awareness related to Superstorm Sandy on December 13, 2012.

## Methodology

Advance notice events (e.g., hurricanes) provide responders with crucial time to better prepare for the implementation of a Twitter monitoring strategy. As it became clear that Sandy would be making landfall somewhere along the eastern seaboard of the US, ASPR analysts began implementing a two-prong monitoring strategy. The strategy included the use of: 1) Twitter lists; a curated list of Twitter users usually tweeting about a single topic^14^ and 2) Boolean searches; using the words *AND, OR, NOT *(Boolean operators) to limit or broaden a search.^15^

These two methods were employed in tandem to retrieve information that addressed an HHS pre-determined list of hurricane Essential Elements of Information (EEIs). The HHS EEIs guide in determining: what information is critical, who is responsible for the data collection, and the frequency of reporting for HHS emergency response operations.^16^ The EEIs cover a variety of public health concerns related to hurricanes and response operations; however, not all of which are likely to be reported on Twitter.

Table 1 includes only the EEI categories that ASPR monitored on Twitter for potential relevant information that would improve HHS situational awareness. The full list of EEI categories is more extensive. Throughout the emergency response, the informational needs and HHS response concerns changed on an almost daily basis as the situation on the ground shifted. Flexibility and adaptability were critical to effectively monitor Twitter for evolving information needs, trends, and situational awareness. Therefore, the monitoring strategy used was driven by two primary factors: changes in key word terminology associated with the event on social media and the informational needs of leadership and field teams. The EEI categories listed in Table 1 were selected for monitoring on Twitter as they were top priorities that had been reported on social media channels during previous incidents.

**Table 1: HHS EEI Categories Monitored on Twitter:** 

Status of critical infrastructure (i.e., hospitals, nursing homes, mental health clinics)
Status of shelters
Property damage in affected area and casualties (including fatalities)
Status of medical special needs populations
Injury/disease surveillance and outbreaks
Mandatory evacuations and relocation assistance
Medical assistance required with Urban Search and Rescue Teams
Environmental conditions (including contamination)
Status of vulnerable populations

HHS and ASPR do not use or rely solely on Twitter for information on any of the EEI categories, as social media (including Twitter) is not always an appropriate source for certain types of information such as the status of deployed personnel or supply needs within medical facilities.

The Twitter list and Boolean searches functioned differently (Figure 1) to filter incoming data from Twitter in order to make it more usable and relevant to the concerns of the ongoing HHS response. The use of the two together provided a more complete and robust picture from Twitter. For example, the Twitter list acted as a wide net with specific sized holes to monitor and capture all EEIs of concern that were openly reported by local news, public officials, and local emergency management who were already reporting on Superstorm Sandy. An added benefit of the Twitter list is that it also captured unknown or unconsidered hazards. In contrast, the Boolean searches used a variety of keywords and limiting or broadening Boolean operators to focus on and extract tweets that included more specific EEIs of concern (e.g., hospital evacuation) from the entire sea of Twitter. On its own, the sole use of a Twitter list would result in a search that was too broad, while using only Boolean searches would lead to results that are too narrowly focused.

**Fishing for Information: Twitter Lists vs. Boolean Searches:**
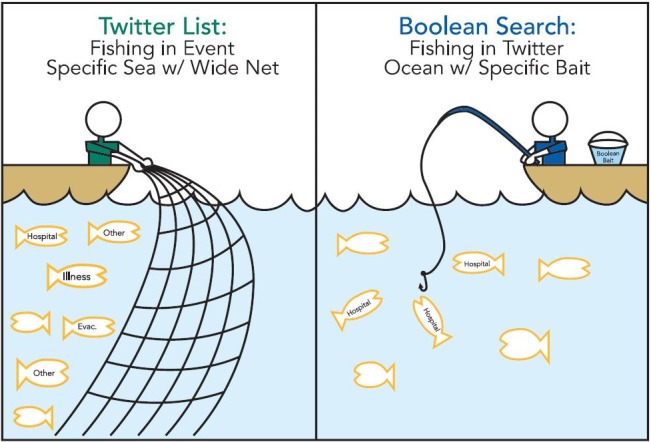

## Development and Use of Twitter Lists

As Bennett and colleagues 2 learned during HHS’s response to Hurricane Isaac, following local public Twitter accounts can result in timelier information, as these individuals and organizations provide first-hand information and on-the-ground reports.^5^ Therefore, for Sandy, the Twitter list developed and used consisted only of local news, reporters, government officials and emergency management accounts. Table 2 lists the types of locale-specific Twitter accounts that comprised the list used by ASPR. Ideally, for an emergency response, a locale-specific Twitter list should at a minimum include these types of Twitter accounts (if they exist).

When the Twitter list was built for an advance notice event (i.e., hurricane), each account was reviewed and verified based on the user’s Twitter profile and Twitter use. Specifically, analysts looked at when the user’s profile was created, how often the user tweets, who is following the user, and historically what kind of information the user tweets. This information was used to verify that real and reliable accounts were added and used. By taking time beforehand to review and verify potential Twitter accounts for a Twitter list, a more trustworthy source of potential information is identified ahead of time thereby allowing analysts to have greater confidence in the information provided via that source during the response.

**Table 2: Locale-Specific Twitter List:** 

• Governor
• State Emergency Management or Homeland Security Agency
• Local/City Emergency Management Agency
• State/Local/City Department of Health
• Local Mayor’s Office
• Local Police Department
• Local News Stations/Local Newspapers
• Local Journalists

Generally, a pre-made Twitter list for an emergency response would focus on one to two major geographic areas impacted by the emergency. However, with Superstorm Sandy, it remained unclear what areas would be hardest hit until just hours before landfall; models varied, with some citing Washington, D.C. and the Eastern Seashore as the area of greatest concern while others were reporting a direct hit to New York City. Due to this uncertainty leading up to landfall, the Twitter list initially developed covered a large area of the East Coast ranging from Virginia to Rhode Island.

However, within the first 24 hours of landfall, major updates were made to the Twitter list as the storm path became clearer. Twitter accounts from states north of Connecticut were culled from the list, and the number of accounts from states south of New Jersey was thinned, but not eliminated entirely. For example, the National Capital Region (Maryland, Virginia, Washington, D.C.) remained a focal point because the HHS headquarters are located within this area.

The focus of the revised and ever evolving Twitter list became New York, New Jersey, and Connecticut. In the first 24 to 48 hours post-landfall, the number of accounts included on the Twitter list from each of these states grew rapidly. Very few of these additions were the result of actively searching for new accounts. Instead, new accounts were added to the Twitter list during active monitoring, usually through indirect recommendations (e.g., retweeting) of existing list members. For example, if a local news station already included on the list retweeted one of its reporters, who was not on the list and who was live-tweeting an unfolding situation, that reporter would then be added to the Twitter list. Throughout the course of Superstorm Sandy, the locale-specific Twitter list not only provided increased situational awareness, but also resulted in a deluge of new sources of information.

As the HHS response grew longer, the number of accounts reporting Sandy-related information decreased to those reporting on, and often from, the most impacted areas. This reinforced what Palen and colleagues learned from the Red River floods, in that interest and attention to a disaster is sustained by those who are local and most impacted by the event.^5^ In the weeks following landfall, the Twitter list was thinned to remove accounts that were no longer reporting Sandy-related information regularly. As accounts were added and removed in an ad-hoc manner during parts of the monitoring throughout the course of the overall emergency response, it cannot be determined how large the list grew to be at any one point. However, when HHS ASPR ceased Twitter monitoring for Sandy, the Twitter list consisted of 77 accounts representing the various categories depicted in Table 2.

## Development and Use of Boolean Searches with Twitter

The initial Boolean Twitter searches created for the HHS Superstorm Sandy response focused on six general areas of concern from the EEIs: hospitals, nursing homes, shelters, injuries/fatalities, cold-related illnesses, and carbon monoxide poisoning. At the beginning, these early subject specific searches were kept simple by using broad hurricane terminology *(Sandy OR Hurricane) *and EEI qualifiers with simple Boolean commands to retrieve storm-related tweets (Table 3). An important note is that while Sandy was not meteorologically a hurricane at landfall, much of the general public still referred to it as a hurricane and for that reason “hurricane” was still used in the search terminology. In addition, because the social media dashboard used by ASPR analysts searched for keywords within hashtags the use of #Sandy or other hashtags was not needed within any of the subject specific Boolean searches. These EEI searches were intentionally broad at the beginning of the monitoring process. Analysts would look for key issues within the results of each EEI search. Narrower and more complex searches were not used to prevent the exclusion of issues that were not identified prior to the response yet are still of importance and interest due to the unique nature of each emergency response.

**Table 3: Initial Boolean Searches Used to Search Twitter:** 

(Sandy OR hurricane) AND hospital
(Sandy OR hurricane) AND "nursing home"
(Sandy OR hurricane) AND shelter
(Sandy OR hurricane) AND (sick OR injured OR death OR dead OR fatality OR killed OR died)
(Sandy OR storm OR hurricane) AND (snow OR cold)
(CO OR "Carbon monoxide") AND (poisoning OR generator) AND (Sandy OR Hurricane)

Abbreviation: CO = Carbon Monoxide

During the course of the response, the Boolean searches were edited to account for over-tweeted information or news. As an example, following multiple hospital evacuations, there was an abundance of stories about nurses evacuating babies in the midst of power outages. As a result, these stories overwhelmed the pre-established hospital search stream. Therefore, to better control the information retrieved, the hospital search was adjusted to exclude the terms most commonly used in the tweets referring to this incident (e.g., “hero” and “babies”). By excluding these terms, the level of “noise” and volume of tweets retrieved via this search was decreased to a more manageable volume. Figure 2 outlines the steps made to adjust the hospital search. These adjustments were made to account for trends within the Twitter conversation.

**Adjusting the Hospital Boolean Search for Trending Terms:**
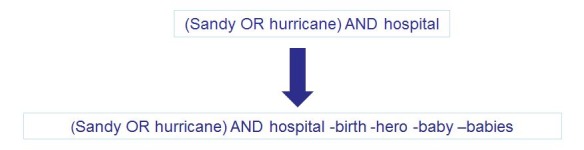

As the storm weakened and moved north into Canada, further edits and additions were made to the Boolean searches to account for changes in the overall Twitter conversation. Analysts found that as more time passed from the date of landfall, Twitter users referred to the storm less by name. People who had regularly been providing updates via Twitter became less likely to use #Sandy and more likely to simply state a problem or concern with the understanding that they were already talking about Sandy.

Figure 3 depicts the results of a retrospective analysis using the Topsy Pro Public Sector Analytics™ (Topsy) of the use of the words *“Sandy” *and *“Hurricane” *on Twitter between October 17, 2012 and November 17, 2012 within the U.S., excluding retweets. As shown, the use of *“Sandy” *and *“Hurricane” *spiked very quickly on October 29^th^, the day of landfall, but almost as quickly began to decrease in use. This did not mean people were no longer tweeting and providing information, but rather that people were tweeting in a different way. Therefore, changes needed to be made to searches to account for the evolving Twitter discussion and to reflect how people now referred to the event.

**Use of “Sandy” and “Hurricane” on Twitter in the United States, October 17, 2012 – November 17, 2012:**
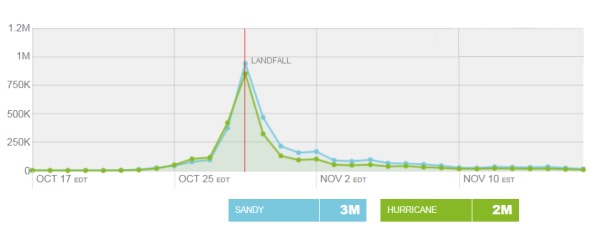

As Twitter is not easily searchable based upon geography and only an estimated 2% of all tweets on a typical day include geographic metadata,^17^ analysts replaced *“Sandy” and “Hurricane”* with basic geographic place names in Boolean searches. For example adding *(NYC OR “New York” OR Brooklyn),* lead to the retrieval of more specific and potentially relevant information, and thereby a better perspective of the situation within the impacted communities. From the previous example of adjusting a Boolean search based upon trending terms (i.e., babies, hero), the additional geographic terms further helped narrow the scope and volume of potentially relevant tweets (Figure 4). Also in addition to adding geographic terminology, analysts chose to further filter the incoming results by adding in the qualifying term of “patients.” This addition helped better focus search results to more relevant tweets referencing the healthcare system.

**Adjusting the Hospital Boolean Search for Shifting Conversations:**
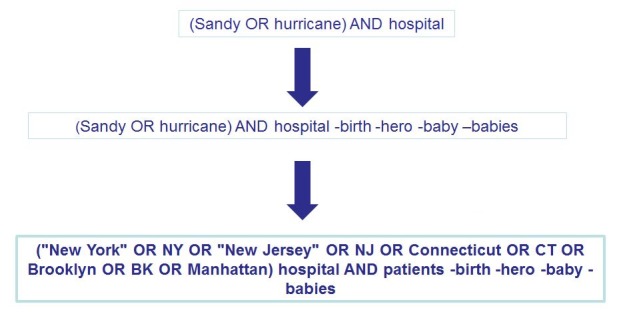

Throughout the HHS response the Twitter list and all Boolean searches were monitored via a social media dashboard to allow for ease of viewing and streaming of information. This dashboard allowed for real-time streaming of filtered data and was essential to organizing searches and incoming information into manageable streams. Each analyst utilized his/her own individual dashboard with identical searches and a subscription to the Twitter list. Edits and changes to the list and searches were managed by the lead analyst (SHS) and communicated and tracked on a single document.

## Two Examples of the Two-prong Strategy in Action

The monitoring of Twitter using the two-prong strategy outlined above involved significant flexibility and adaptation. When Twitter monitoring was ended for the Sandy response there were a total of 20 Boolean searches followed regularly with additional ad-hoc searches created as needed for further information. The use of this prong of the strategy resulted in the retrieval and flagging of multiple EEI events during the course of the Sandy response. Throughout the HHS response, more than 30 Twitter reports or responses to information requests in addition to daily summaries were sent.

One example that demonstrated the value of developing and using a dynamic Twitter monitoring strategy to enhance situational awareness during Superstorm Sandy was the evacuation of Bellevue Hospital in New York City.

***Boolean Searches and Bellevue Hospital***

The use of Boolean searches to track the status of healthcare infrastructure was valuable during the HHS response. The evacuation of a major hospital places additional stress on state and local health systems; therefore, early knowledge or awareness of an evacuation (or potential for) could potentially help HHS better prepare beforehand should states request federal assistance. Boolean searches worked well for this EEI category as it was a narrow subject matter with defined concerns. Around 8:45 p.m. EST on October 29, 2012, the standard hospital Boolean search retrieved tweets that the Bellevue Hospital was operating on emergency generators (Figure 5). Using the information provided in the initial tweets, new and more specific Boolean searches were created using “Bellevue” as a keyword to attempt to retrieve further details and enhance situational awareness.

After the creation of more targeted Boolean searches it was determined that Bellevue Hospital’s emergency generators were not working properly and the basement was flooding with water. Bellevue Hospital hovered in a critical situation before fully evacuating its final 500 patients nearly two days later. This example demonstrated that while it was not plausible to monitor every facility or potential incident of concern ahead of time (particularly in high population density locations), the use of broad searches to identify defined topics of concerns with further investigation as necessary has the potential to be a more efficient use of time and energy during a response.

**The Bellevue Hospital Evacuation Told Through Twitter:**
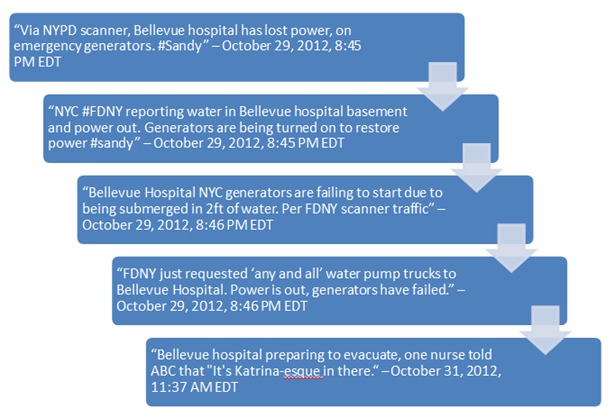

***Twitter List and Norovirus***

Some EEIs were more easily tracked via the Twitter list due to the broad nature of the subject matter. Disease surveillance and outbreaks is a broad subject that could encompass a variety of different illnesses and concerns. It would have been too difficult to attempt to predict and create Boolean searches for every possible illness that could emerge as a consequence of the storm and subsequent recovery efforts. Instead, the Twitter list was used as a wide net to monitor overall community health in the days, weeks, and months following the storm.

For example, on November 6, 2012, a local New York newspaper tweeted a report of an outbreak of norovirus at an evacuee shelter. This was the first report ASPR retrieved from Twitter regarding gastrointestinal illness outbreaks at evacuee shelters. By capturing this first mention of a disease outbreak via pre-identified source on Twitter and disseminating this information to HHS personnel, HHS field staff increased precautions and was better prepared to mitigate similar outbreaks in facilities where NDMS representatives and USPHS officers were located.

## Limitations and Lessons Learned

There were several limitations associated with this method of using Twitter for situational awareness. The ability to verify and fully trust information obtained from Twitter and other forms of social media was a concern. Fake Twitter accounts and rumors can quickly inundate Twitter following a disaster. For example, following the Boston bombings in 2013, 29% of the most tweeted content was the result of rumors and fake accounts.^18^ Superstorm Sandy was no exception with reports quickly spreading on Twitter of the flooding of the New York Stock Exchange, sharks swimming in the New Jersey streets, and scuba divers in the New York City subway. The rise in popularity of graphic design and photo editing programs has led to an increase in visual rumormongering. Gupta and colleagues,^19^ stated that during Superstorm Sandy 10,350 tweets circulated on Twitter that contained fake images; 86% of these were retweets. As a result of these two examples, good practice would entail not using or trusting an image tweeted or retweeted, unless its original source could be verified.

It was critical to have a verification system in place prior to the activation of monitoring in order to avoid perpetuating the spread of rumors and inaccurate information.^20^ Tweets found through the Boolean searches were approached with greater caution than those found via the pre-established Twitter list. The Twitter list was comprised of pre-verified accounts thereby increasing its veracity and reliability; however, even tweets from these sources were reviewed carefully. No matter how the tweet was retrieved, if a Twitter source provided questionable information, that information would not be reported on until it could be corroborated or verified by additional sources.

However, during the early hours of the HHS response, the verification process used to add accounts to the Twitter list was modified due to significant constraints on time and resources. The modified process focused on the Twitter account profile information such as the profile biography, creation date, number of followers, and number of tweets sent. Unlike during the preparation phase, a review of past tweets sent by the user was not performed.

The level of situational awareness gained from the EEI Boolean searches was only as good as the keywords or phrases they contained. Building good searches required an understanding of the vocabulary associated with the ongoing response and EEIs of concern. It also required a familiarity with the vernacular of the impacted population and geographic area. Analysts had to be willing to immerse themselves in the conversation to gain a better understanding of the needs of the impacted population.

Nonetheless, overdependence on Boolean searches could lead to the exclusion of certain tweets that may contain important information not included in the search terminology. It is important to acknowledge that every event is unique, and that there inevitably will be a certain number of unknown factors that do not fall into the predetermined EEI categories. Broad searches allow room for anomalies or outliers. The search,*(sandy OR hurricane) AND hospital*, is made unique enough by the term hospital to filter out a majority of Sandy related tweets while still limiting the return to tweets within the public health sector. The simplicity of this search also limits the need to account for spelling errors, abbreviations or differences in vernacular within tweets. For example, a known concern was generator failure at hospitals. If the following search had been constructed: *(sandy OR hurricane) AND hospital AND (generator OR “lost power”), *any tweet containing a misspelling or alternate phrasing such as “*NYU Hospital is **without power**.* *Don’t think their backup **gen**. is working*” would not be retrieved. Until a more specific concern was established (i.e., the status of Bellevue Hospital’s generator) it was more beneficial to maintain broad searches with minimal keywords.

As stated earlier, hashtags were not used in the initial set of Boolean searches as the social media dashboard used included hashtags in the search results. However, a short and simple search was created to track the traffic of a few major hashtags (i.e., *#Sandy OR #Snoreastercane OR #Frankenstorm)*. But, the lack of limiting terminology (e.g., *hospital *or*“nursing home”*) resulted in a flood of tweets retrieved via this Boolean search. Additionally, as these hashtags began to trend or increase in popularity and usage, they were used as tweet spam whereby they contained no information relevant to Sandy. Therefore, the absence of EEI terms and the addition of Twitter spam made this stream virtually useless.

This two-prong monitoring strategy required a staff completely dedicated to monitoring and managing the Twitter list and Boolean searches. Staff limitations were a factor for Twitter monitoring during Superstorm Sandy due to the number of team members deployed as part of the HHS response. In the U.S., emergency managers report shortages of personnel as the number one reason why their local agencies do not incorporate social media in their emergency management activities.^21^ The use of additional ad-hoc HHS employees and interns provided an increase in numbers, but resulted in an initial loss in man-hours while new team members were trained for monitoring. Ideally, the use of ad-hoc staff for Twitter monitoring would involve some type of pre-training or improved just-in-time training to allow for a more efficient transition and retrieval of relevant results.

The use of Twitter varies demographically, geographically, and even politically, making the use of Twitter for each incident or event different. As such, every disaster is different and the same search techniques and strategies may not be applicable and relevant for every response. However, having a starting point of pre-established Boolean search strategies based on existing information needs and priorities, a process for verifying Twitter accounts immediately before and during response, and documenting changes made along the way are crucial to making Twitter work for augmenting situational awareness.

## Conclusion

The most important lesson learned from Superstorm Sandy was the need for a dynamic and flexible monitoring process and strategy to understand and respond quickly to health needs in the areas impacted by Superstorm Sandy. Search strategies should change as frequently as the unfolding event. The inability to adapt to a changing situation ensures stale and stagnant terminology and search results. Twitter lists and Boolean searches should be used together to maximize situational awareness. The most important information comes from the impacted population, whether news, local government or local citizens. These are the people who care the most about the information being disseminated. The low number of geo-located tweets and inability to filter tweets based on location in many tools can present a problem in locating tweets from directly impacted areas. Therefore, the use of a targeted Twitter list can help to overcome this hurdle while simultaneously providing more trustworthy content. This information can often be used to guide what additional Boolean searches should be created to fish for individual tweets that provide additional details.

In addition, each disaster presents its own unique hazards and challenges. An understanding of the impacted populations and communities will allow for the development of a more thoughtful and complete Twitter monitoring strategy. This includes taking into account the demographics of the impacted area, whether the area is urban or rural, and how active local government is on Twitter. Each of these will have a major impact on the amount and type of information that is available via Twitter throughout the course of the response.

The use of a two-pronged approach to monitoring Twitter during Superstorm Sandy proved beneficial to the HHS response and augmented overall situational awareness of the affected areas. The strategy used accounted for known (i.e., EEI Boolean searches) and unknown informational needs (i.e., Twitter list). Yet, it is not enough to create a Twitter list and some Boolean searches at the beginning of an event and expect a consistent return of useful information. Twitter monitoring during an event involves constantly reacting to trends in the conversation and changing informational needs, and must remain fluid and flexible to prevent the retrieval of irrelevant information resulting from stagnating searches and lists. Investing in the technology, personnel, and training needed to effectively and efficiently monitor Twitter during a response has the potential to result in a high return on investment and inform on the needs of impacted communities.

## Footnote

*At time of writing. The number of active monthly users and tweets sent each day is a fluid number.

## Competing Interests

The authors have declared that no competing interests exist.

## References

[1] Sarter NB, Woods DD (1991) Situation awareness: A critical but ill-defined phenomenon. The Int J Aviat Psychol 1: 45-57.

[2] Bennett KJ, Olsen JM, Harris S, Mekaru S, Livinski AA, et al. (2013) The perfect storm of information: combining traditional and non-traditional data sources for public health situational awareness during hurricane response. PLoS Curr 5. Edition 1. doi: 10.1371/currents.dis.d2800aa4e536b9d6849e966e91488003. 10.1371/currents.dis.d2800aa4e536b9d6849e966e91488003 PMC3871418 24459610

[3] Twitter: About. Available: https://about.twitter.com/company. Accessed: March 10, 2014.

[4] NBC News (November 12, 2013) Typhoon Haiyan: Filipinos use social media to ensure no victim goes unaided. Available: http://worldnews.nbcnews.com/_news/2013/11/12/21416986-typhoon-haiyan-filipinos-use-social-media-to-ensure-no-victim-goes-unaided?lite. Accessed: March 10, 2014.

[5] Palen L, Starbird K, Vieweg S, Hughes A (2010) Twitter-based information distribution during the 2009 Red River Valley flood threat. Bull Am Soc Inf Sci 36: 13-17.

[6] Sutton J, Palen L, Shklovski I. Backchannels on the front lines: emergent uses of social media in the 2007 southern California wildfires. Proceedings of the 5th International ISCRAM Conference; May 2008; Washington, DC. pp. 9. Available: http://www.jeannettesutton.com/uploads/BackchannelsISCRAM08.pdf Accessed: July 10, 2014.

[7] Gilgoff D, Lee JJ (April 15, 2013) Social media shapes Boston bombings response. National Geographic News. Available: http://news.nationalgeographic.com/news/2013/13/130415-boston-marathon-bombings-terrorism-social-media-twitter-facebook/ Accessed: March 10, 2014.

[8] Greene CH, Francis JA, Monger BC (2013) Superstorm Sandy: A series of unfortunate events? Oceanography 26: 8-9.

[9] Blake ES, Kimberlain TB, Berg RJ, Cangialosi JP, Beven JL (February 2013) Tropical Cyclone Report Hurricane Sandy (AL182012) 22 – 29 October 2012. Miami, FL: National Hurricane Center. 157 p. Available: http://www.nhc.noaa.gov/data/tcr/AL182012_Sandy.pdf. Accessed: April 7, 2014.

[10] U.S. Department of Homeland Security (May 2013) National Response Framework. Washington, DC: U.S. Department of Homeland Security. Available: http://www.fema.gov/national-response-framework. Accessed: May 1, 2013.

[11] U.S. Department of Health and Human Services, Office of the Assistant Secretary for Preparedness and Response (December 4, 2012) Hurricane Sandy - Public Health Situation Updates. U.S. Department of Health and Human Services Hurricane Sandy Response and Recovery Activities. Available: http://www.phe.gov/newsroom/Pages/situpdates.aspx. Accessed: March 10, 2014.

[12] Tweet from @Twitter – sent November 2, 2012 at 12:46 EDT.

[13] Tweet from @Twitter – Sent November 2, 2012 at 12:48 EDT.

[14] Twitter, Inc. Using Twitter Lists. Available: https://support.twitter.com/articles/76460-using-twitter-lists#. Accessed: April 7, 2014.

[15] Katz W (2002) Introduction to Reference Work: Basic Information Services. New York, NY: McGraw-Hill.

[16] U.S. Department of Health and Human Services, Office of the Assistant Secretary for Preparedness and Response (July 2010) ESF #8 2010 Hurricane Playbook. Washington, DC: U.S. Department of Health and Human Services. pp. 110.

[17] Leetaru K, Wang S, Cao G, Padmanabhan A, Shook E (May 2013) Mapping the global Twitter heartbeat: The geography of Twitter. First Monday 18. Available: http://firstmonday.org/ojs/index.php/fm/article/view/4366/3654 Accessed: March 10, 2014.

[18] Gupta A, Lamba H, Kumaraguru P. $1.00 per RT #BostonMarathon #PrayForBoston: Analyzing Fake Content on Twitter; Proceedings of the Eighth APWG eCrime Research Summit (eCRS); 2013; San Francisco, CA. pp. 12. Available: http://precog.iiitd.edu.in/Publications_files/ecrs2013_ag_hl_pk.pdf Accessed: July 10, 2014.

[19] Gupta A, Lamba H, Kumaraguru P, Joshi A (2013) Faking Sandy: characterizing and identifying fake images on Twitter during Hurricane Sandy. Proceedings of the 22nd International Conference on World Wide Web Companion. Rio de Janeiro, Brazil: International World Wide Web Conferences Steering Committee. pp. 729-736. Available: http://dl.acm.org/citation.cfm?id=2488033 Accessed: July 10, 2014.

[20] (2014) Verification Handbook; Silverman, C. (Ed.). Maastricht: European Journalism Centre. 122 p. Available: http://verificationhandbook.com/downloads/verification.handbook.pdf. Accessed: 14 April 2014.

[21] Hiltz SR, Kushma JA, Plotnick L. Use of social media by U.S. public sector emergency managers: barriers and wish lists. 11th International Conference on Information Systems for Crisis Response and Management; 2014; Penn State University, University Park, PA, USA. Available: http://iscram2014.ist.psu.edu/sites/default/files/misc/proceedings/p11.pdf Accessed: July 10, 2014.

